# Development of smart patient care robot with enhanced autonomous navigation through path optimization in hospital wards

**DOI:** 10.1038/s41598-026-36664-2

**Published:** 2026-01-22

**Authors:** Bumsoo Kim, Jaeho Hyun, Bomi Yang, Youngjin Moon, Jaesoon Choi

**Affiliations:** 1 Biomedical Engineering Research Center, Asan Medical Center, Asan Institute for Life Sciences, Seoul, 05505 Republic of Korea; 2 Department of Biomedical Engineering, University of Ulsan College of Medicine, Seoul, 05505 Republic of Korea

**Keywords:** Healthcare, Wheeled mobile robot, Autonomous driving algorithm, Occupancy grid map, Enhanced D* lite, Navigation success rate, Biomedical engineering, Mechanical engineering

## Abstract

This study presents the development of a smart patient-care robot designed to assist healthcare workers by automating routine monitoring tasks and enhancing autonomous mobility in hospital wards. Unlike conventional systems that rely on multiple sensors, the proposed platform achieves reliable navigation using a single two-dimensional light detection and ranging sensor combined with an enhanced D* lite algorithm. A key contribution of this work is the introduction of virtual obstacle spaces in occupancy grid maps, which simplify path planning and significantly increase the success rate of passing through narrow doorways, one of the most collision-prone scenarios in ward environments. Experimental results show a navigation success improvement of over 30%, confirming enhanced safety and efficiency in mobility. This research establishes a practical foundation for deploying patient-care robots in real hospital settings, showcasing both technical advances in autonomous navigation and the potential to reduce healthcare staff workload while ensuring patient safety.

**Supplementary Information:**

The online version contains supplementary material available at 10.1038/s41598-026-36664-2.

## Introduction

 There has been a rapid increase in the daily use and demand for robotics and automation in modern healthcare^1^. Autonomous mobile robots have already been deployed in hospital logistics, suggesting the potential for broader integration with patient-care systems^2^. Currently, the healthcare system is under significant strain because of the increase in aging populations, chronic diseases, and patients with infectious diseases^3,4^. The physical and mental well-being of healthcare workers such as nurses are essential for ensuring safe and high-quality medical services^5,6^. A recent study conducted across six countries emphasized the relationship between nurse burnout and healthcare service quality, thereby highlighting the importance of addressing the well-being of nurses in the healthcare system^7,8^. According to Mo et al.^9^, the COVID-19 pandemic exacerbated these issues by subjecting nurses to increased workloads, exposure to viruses, and the emotional stress of caring for patients in high-risk environments. Recent literature highlights the rapid advancements in AI robots, particularly in direct patient care, with the development of systems like the automated robotic CPR system and sputum suction robot that offload strenuous physical tasks from medical staff^10^. These robots, along with socially aware and visiting robots that enhance patient-family interaction and psychological support, have been extensively deployed in hospital settings, especially during the COVID-19 pandemic, proving their value in reducing infection risks and improving operational efficiency.

To address these problems, robotic technology has received increasing attention, particularly as an innovative tool for healthcare and welfare services^11^. Recent surveys highlight the wide applicability of healthcare robots in hospitals, ranging from patient monitoring to assistance in daily tasks^1^. In addition, nursing officers emphasize the importance of autonomous mobile robots in reducing non-nursing duties and improving patient safety^12^. Recent studies have also demonstrated the use of mobile robots to perform disinfection tasks in hospitals to prevent disease transmission^13^.

Recent studies on mobile robot navigation have focused on advanced technologies and applications, including LiDAR, indoor GPS, and deep reinforcement learning in autonomous navigation systems^14–17^. Path planning is essential for enabling autonomous navigation and optimizing the efficiency of robotic systems. Various approaches have been developed to address challenges such as navigating cluttered environments, improving energy efficiency, and maintaining precision across diverse terrains^18^. However, to the best of our knowledge, no attempts have been made to create a safer environment through the use of virtual obstacle spaces. This study aims to present a novel approach to improving autonomous navigation stability through the implementation of virtual spaces.

Route optimization, which is a key element in addressing these challenging tasks, includes algorithms and strategies that enable robots to minimize unnecessary movements while efficiently and safely reaching their destinations. This study explored the potential of a mobile robot system to assist nurses in quickly identifying events, such as falls or high-fever patients, especially during vulnerable hours in hospital wards, thereby enabling a prompt response within the golden time to prevent accidents. To achieve this, the trajectory of the robot was discretized into single-pixel units, and the surrounding terrain was modeled more conservatively than in reality, reducing potential risks in the actual navigation path. Furthermore, mobile robots equipped with a camera system and omnidirectional bending mechanism can measure patient body temperature, heart rate, and oxygen saturation, triggering alarms to alert nearby personnel or caregivers in hazardous situations.

As a first step, a test was conducted to improve autonomous driving performance through route optimization. The mobile robot used in this study is a smart patient-care robot currently under development in my laboratory. This paper proposes an optimized grid map for a hospital environment using a particle filter and an enhanced D* Lite algorithm-based driving model. The study focused on improving the driving success rate of mobile robots by modifying the occupancy grid map (OGM) used in simultaneous localization and mapping.

The primary contributions of this study are summarized as follows:

1. Development of a mobile platform applicable to real hospital environments by integrating a mobile base (Omorobot R1) with upper modules such as 2D and 3D LiDAR.
2. Enhancement of navigation and path optimization through the implementation of a reliable enhanced D* Lite algorithm and modified OGM, improving door-passage success rates—one of the most collision-prone scenarios—by over 30%.
3. Establishment of an environment for optimal path planning by scaling and calibrating hospital floor plans based on OGM representation.

By modifying the OGM, the robot’s movement route was simplified and guided along safer paths while eliminating unnecessary motions^19^. As a result, operational time was extended by reducing energy consumption and allowing movement along the shortest path. Finally, an optimized path was tested in a real hospital environment, and the driving success rate was evaluated. The paper concludes with an assessment of the practicality of the proposed approach and suggestions for future research and improvements.

## Literature review

Robots are physically embodied systems that can sense and respond to their environment through physical interactions, perform repetitive activities with high precision, and remain unaffected by psychological fatigue^20,21^. Juang and Lo^22^ integrated wheeled mobile robots (WMRs) with charge-coupled device camera (CCD) cameras and ultrasonic sensors to track moving objects. Arjon Turnip et al.^23^ developed a medical assistant robot for use in patient isolation rooms, enabling autonomous movement, interaction, and face detection. Rivai et al.^24^ introduced a two-dimensional (2D) mapping technique using an omnidirectional moving robot equipped with light detection and ranging (LiDAR), while Hai Wang et al.^25^ proposed a real-time vehicle detection algorithm combining vision and LiDAR point clouds. The medical assistant robot by Zhao^26,27^ uses a line-following system, though this is difficult to implement in hospitals due to the need for obstacle-free paths. Seder et al.^28^ integrated the D* search algorithm with a dynamic-window-based local obstacle avoidance algorithm for implementing a control technique for a collision-free mobile robot with guaranteed real-time performance. The D* lite algorithm studied by ‘Koenig & Likhachev’ and ‘Koenig & Maxim’ et al.^29,30^ is one of the most popular goal-directed navigation algorithms and widely used for navigation in unknown environments. An autonomous mobile robot is a system that operates in an unpredictable and partially unknown environment. This means the robot must have the ability to navigate without disruption and having the capability to avoid any obstacle placed within the confinement of movement^31^. A high autonomous driving performance is required for mobile robots to operate effectively in real-world environments. In complex spaces such as hospitals and nursing homes, challenges such as obstacle avoidance, path planning, and real-time environmental awareness remain significant. Obstacle avoidance algorithms are key to enabling safe, efficient navigation while minimizing collision risks^32^. Lakshmi et al.^33^ proposed an autonomous nursing robot system designed for deployment in smart hospital environments. The proposed robot integrates BLE beacon-based localization for patient room identification with multifunctional capabilities including medication delivery, vital sign monitoring (temperature and heart rate measurements), and voice-based patient interaction.

D* Lite is an incremental path planning algorithm that adapts LPA* (Lifelong Planning A*) to robotic navigation, enabling efficient replanning by updating only the affected portions of the path when environmental changes occur, rather than recomputing the entire trajectory from scratch. The algorithm maintains two key values for each node: $\:g$(the current best-known cost estimate from the start) and $\:rhs$ (the one-step lookahead value), while the priority queue employs a two-element key structure that incorporates heuristic information. Whereas the original D* Lite formulation uses simple Euclidean distance as the sole cost metric for g-value computation, our Enhanced D* Lite integrates additional cost terms into the objective function, including risk costs derived from potential fields and rotational costs penalizing heading changes. This multi-term cost function yields paths with significantly improved quality metrics: increased average clearance from obstacles and reduced frequency of sharp turns, thereby enhancing traversal stability and safety margins. Furthermore, while the original D* Lite exhaustively explores all adjacent nodes during shortest-path search, Enhanced D* Lite employs algorithmic constraints on the search space through dynamic range limitation or directional angle restrictions, effectively pruning unnecessary node expansions. Empirically, this selective expansion strategy reduces the average number of expanded nodes during replanning episodes in identical environments, resulting in measurable reductions in computational time. From a heuristic perspective, the original D* Lite relies on a fixed heuristic function, whereas Enhanced D* Lite implements a dynamic weighted heuristic that adaptively modulates weighting factors based on local obstacle density. This adaptive heuristic formulation demonstrably improves path generation success rates in complex, cluttered environments where conventional fixed-weight approaches may fail to find feasible solutions.

## Method and robot

### Smart patient-care robot (SPCR)

**Fig. 1:**
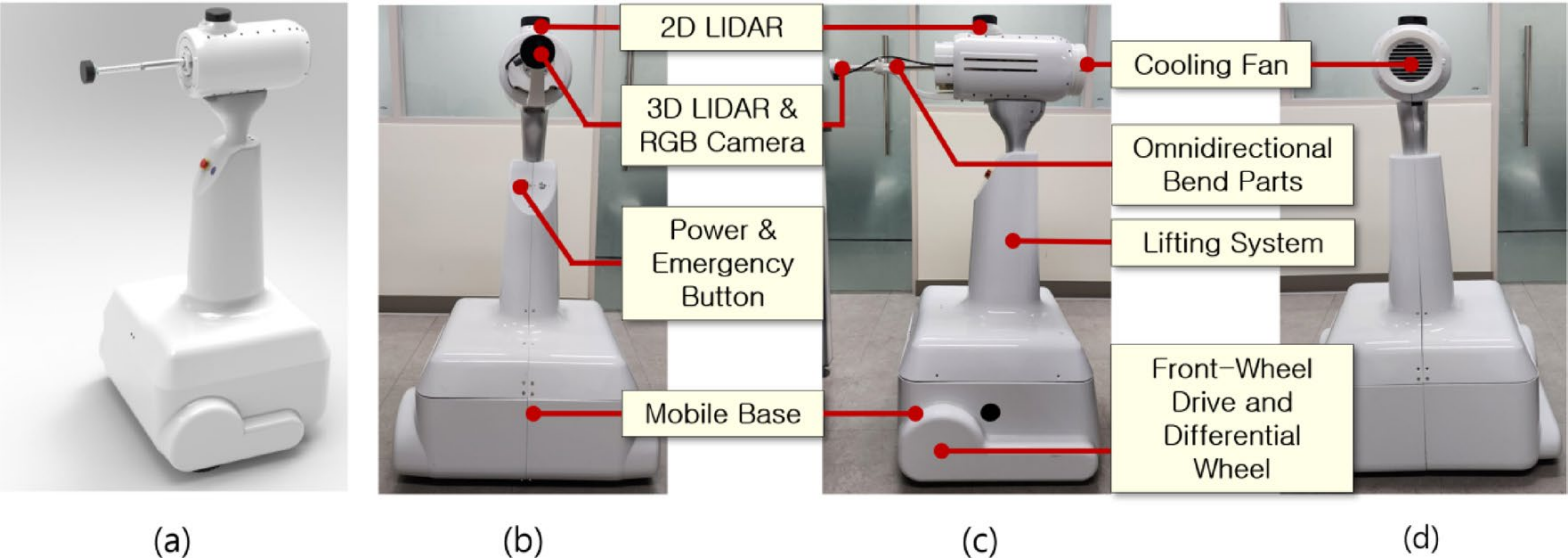
Overview of a smart patient-care robot. (**a**) smart patient-care robot, (**b**) front view, (**c**) side view, and (**d**) rear view.

A smart patient-care robot (SPCR) was designed to effectively implement the required functions while ensuring that sensor performance is not restricted by placement. A three-dimensional (3D) LiDAR camera (L515, Intel Realsense, USA) was installed at the front of the robot to recognize fall incidents and enable non-contact heart rate measurements. A 2D LiDAR (TG30, YDLidar, China) was mounted at the top of the structure to provide a 360° field of view, detect surrounding objects, and output real-time position data for both dynamic and static obstacles. In addition, a cooling-fan-based air circulation system was installed at the rear of the robot to dissipate the heat generated by multiple motors. Figure 1 shows the appearance of the SPCR.

A mobile application control unit equipped with 3D LiDAR, 2D LiDAR, and motion-control components were mounted on the upper part of the SPCR. Six direct-current (DC, Maxon) motors were used to secure the omni-directional movement, including four-axis front bending, one-axis rotation, and one-axis forward/backward movement motors.

**Fig. 2:**
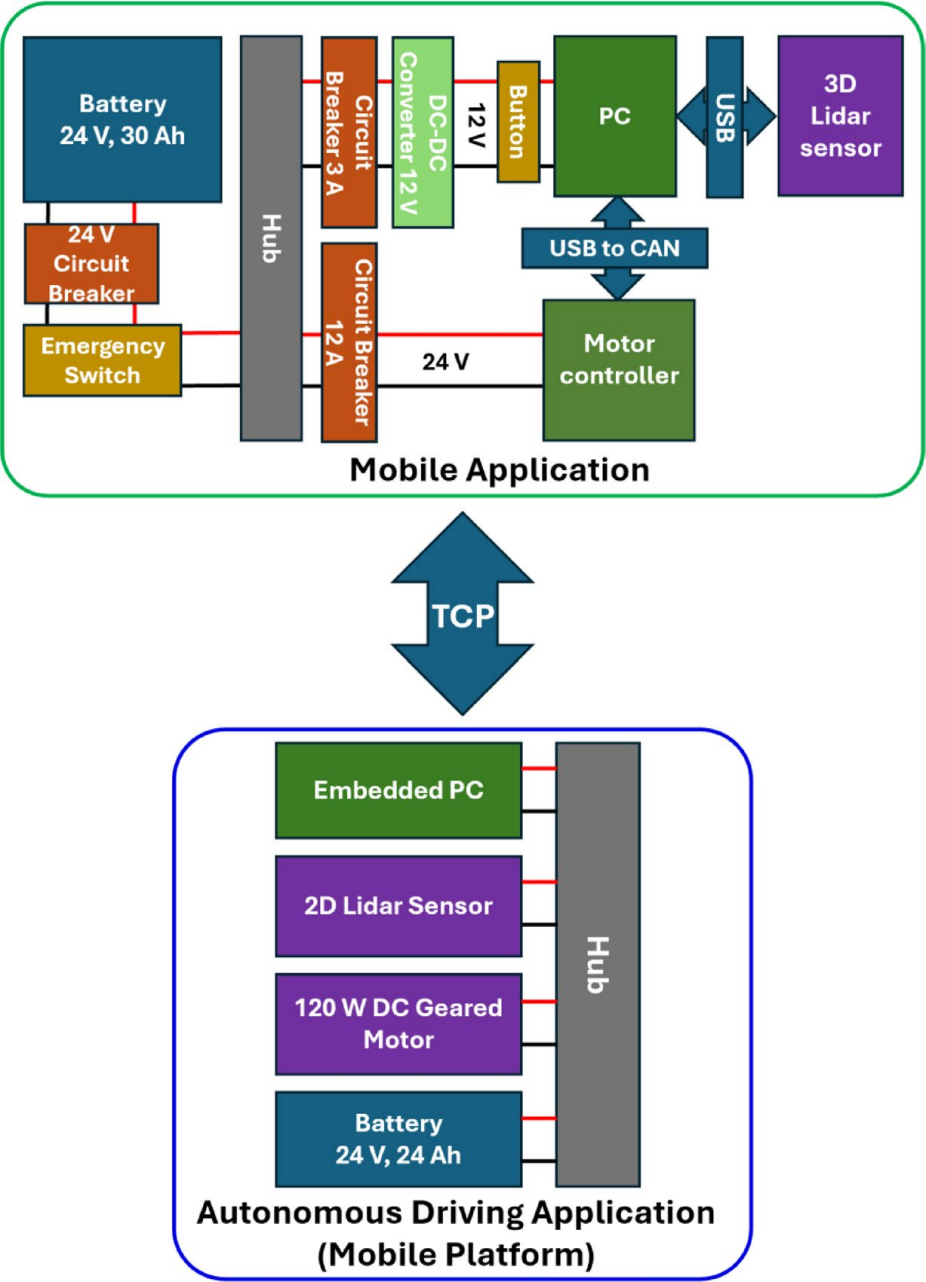
The overall system configuration of the SPCR.

The main control system of the SPCR is structured as shown in Fig. 2: an embedded PC (Intel NUC Core i7, 32 GB RAM) and a battery (Li-ion, 25.9 V, 30 Ah), several types of converters and breakers, and communication devices. The SPCR system included a mobile base configured with a mobile robot (R1, Omorobot, South Korea). The mobile application shown on the left side of Fig. 4 is equipped with a motor control system and 3D LiDAR for omnidirectional movement, fall detection, and heart rate measurement. The autonomous driving application uses 2D LiDAR and an enhanced D* lite algorithm to manage navigation and base movement. The two systems were connected via TCP communication, and a circuit breaker ensured system safety by regulating the required current. Separate batteries were employed to mitigate rapid depletion resulting from the high power demands of autonomous driving applications.

The mobile robot base uses a front-wheel differential structure and it communicates motor-control signals via serial communication. The main parameters of the mobile robot are listed in Table 1^34^.

**Table 1:** Main parameters of the mobile robot.

Parameter	Value
Speed [m/s]	1.2
Load capacity [kg]	100
Handle slope [%]	13
Motor power [W]	120
Operation time [h]	8
Wheel diameter [cm]	20.32
Motion control frequency [Hz]	10
Total weight including battery [kg]	37

### Autonomous driving algorithm

The SPCR must travel from a starting point to a target point while avoiding obstacles. Most mobile robots can navigate through both known and unknown environments^28,35^, and thus must plan the shortest possible path and re-navigate quickly if new obstacles appear^36,37^. In this context, static obstacles refer to those in a fixed environment, while dynamic obstacles are those whose positions change over time.

The most widely used autonomous navigation algorithms are the A* and D The dynamic window approach (DWA) has also been applied to enable socially aware navigation in medical robots^38^. The A* algorithm determines a path using distance and path-cost functions, whereas the D* algorithm enables real-time path re-planning and updating in both static and dynamic environments^39,40^. The navigation algorithm used in this study was an enhanced D* Lite algorithm, which operated on the same principles as the original D* Lite but with improved performance. Unlike A*, which reconstructs a new map and finds a new path whenever the environment changes, D* performs localized path planning based on an already planned global path, enabling real-time path modifications.

**Fig. 3:**
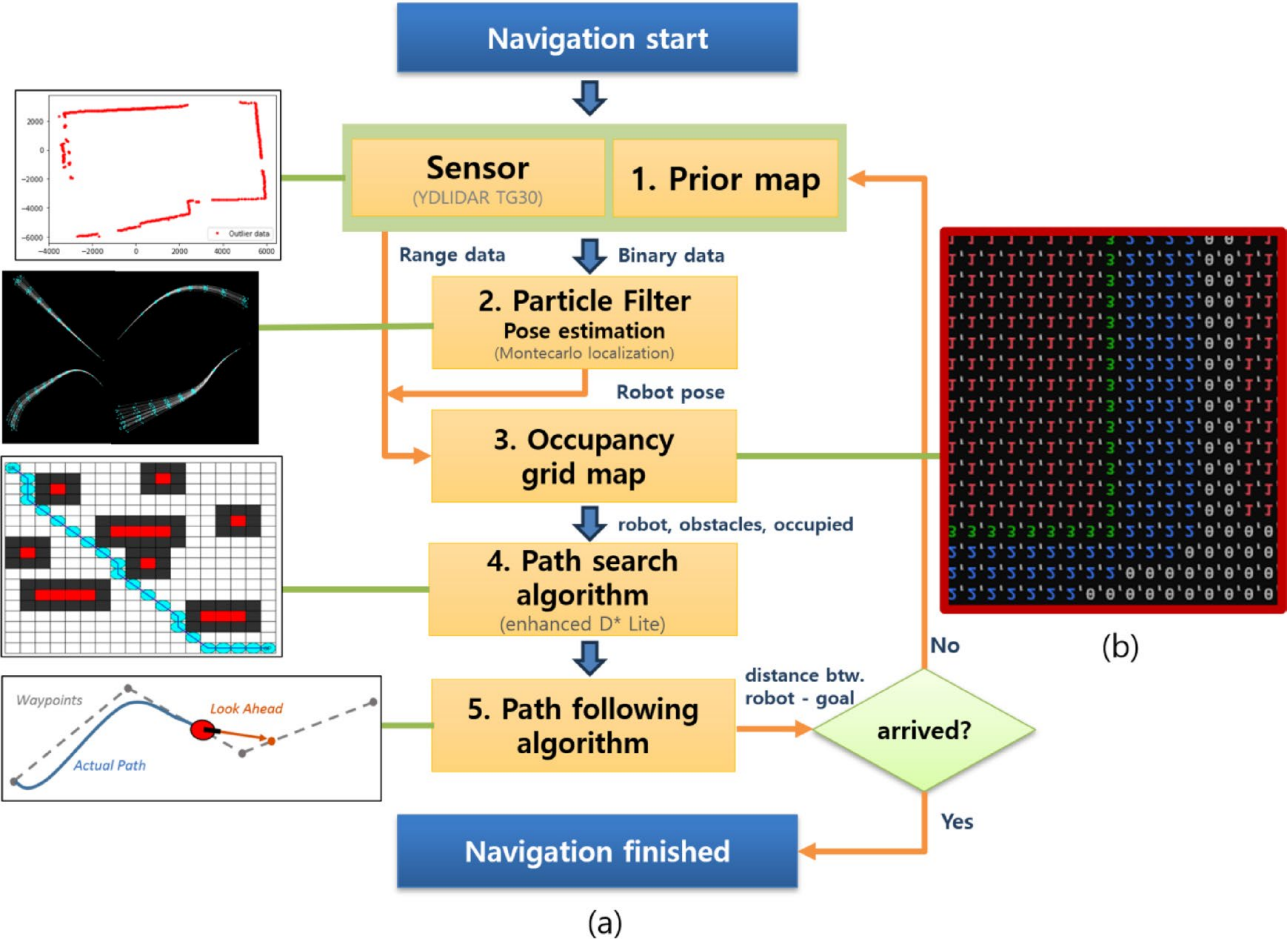
Flowchart of the procedure employed for navigation. (**a**) flowchart and (**b**) numbered map.

In this study, the enhanced D* lite algorithm aimed to maximize navigation success by incorporating modified maps into the occupancy grid map (OGM). A mobile robot must achieve a 100% driving success rate while navigating collision-free paths from start to goal. To achieve this, additional sensors such as LiDAR, ultrasonic sensors, displacement sensors, and cameras are often employed. This study explored maximizing navigation success based only on OGM before adding multiple sensors. A flowchart of this process is shown in Fig. 3.

### Environment modeling

Environment modeling plays a critical role in autonomous navigation, as it directly impacts path planning. The modeling method affects computational time, safety, and the simplicity of the generated path^41^. In this study, the OGM was modified prior to path generation to guide the SPCR along a safe and simplified route on a pre-existing map. Particular attention was given to achieving a high pass rate for standard hospital doors, approximately 900 mm wide, using only 2D LiDAR data. The SPCR, which is 760 mm wide, has about 70 mm clearance on each side when passing through such doors. This narrow space increases the likelihood of collisions, especially when cumulative localization errors occur.

Drawing work for the partial space in the hospital was performed using a distance measurement tool to generate a DXF file. These drawing files were uploaded using a C + + program, visualized in OGM, and adjusted through scaling and calibration to match the sizes of the SPCR and 2D LiDAR. The generated OGM was divided into numbered sections to proceed with the autonomous driving algorithm, as indicated in Fig. 3b, 0 (gray), 1 (red), 2 (blue), and 3 (green) represent the navigable area, non-navigable areas, path-setting zones, and actual navigation path of the SPCR.

### Pose estimation using particle filter

**Fig. 4:**
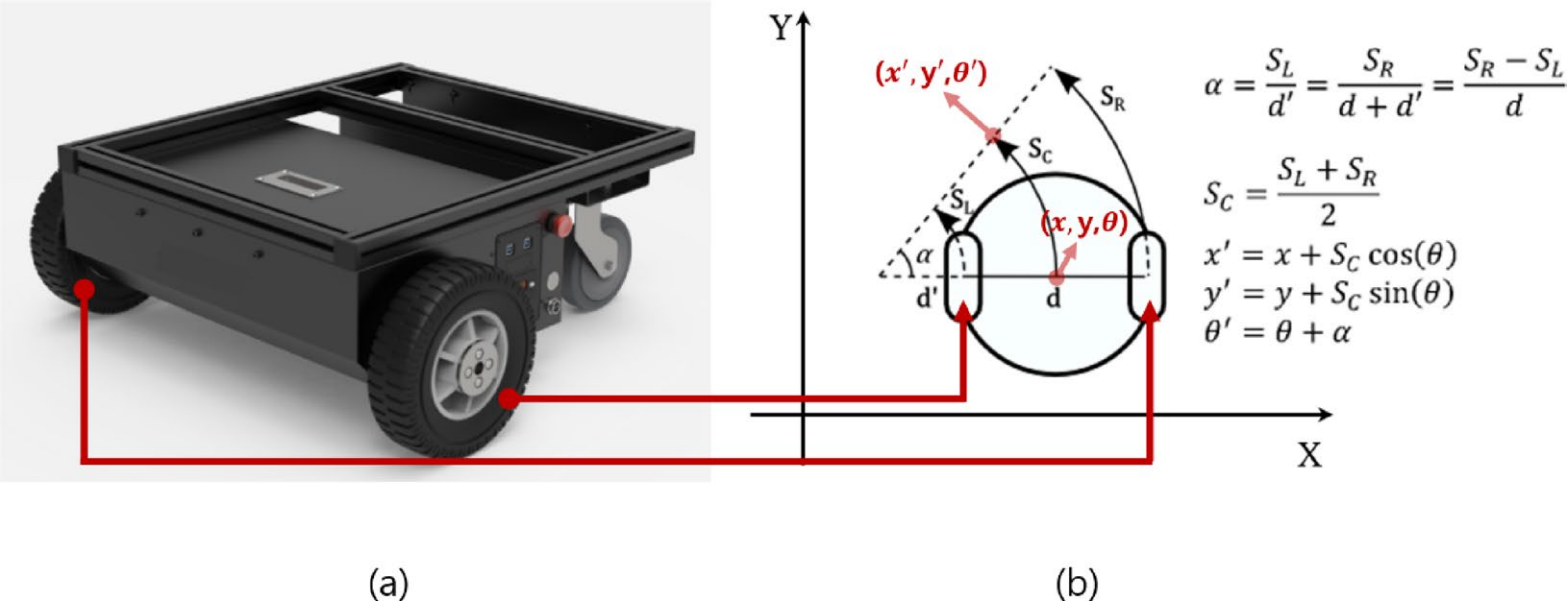
The trajectory analysis based on wheel odometry. (**a**) robot’s front wheel and (**b**) calculation of the trajectory.

The current position of the SPCR is predicted using wheel odometry data, as shown in Fig. 4, and the matching information between the 2D LiDAR and the line map^42^. The main parameters related to the trajectory are listed in Table 2.

**Table 2:** Main parameters related to the trajectory.

Parameter	Description
$\:(x,\mathrm{y}$,$\:\theta\:)$	The robot’s two-dimensional position (x, y) and heading (θ, orientation) before moving
($\:{x}^{{\prime\:}},\mathrm{y}^{\prime\:}$,$\:\theta\:^{\prime\:}$)	The robot’s two-dimensional position ($\:{x}^{{\prime\:}},\:\mathrm{y}{\prime\:}$) and heading ($\:\theta\:{\prime\:}$, orientation) after moving
d	The distance between the wheels of the robot
$\:\mathrm{d}^{\prime\:}$	The distance between the left wheel and the center of rotation that occurs when the robot rotates
α	The angle between the robot’s current position and its next position
$\:{S}_{R}$	Right wheel’s length (arc length)
$\:{S}_{L}$	Left wheel’s length (arc length)
$\:{S}_{C}$	The average distance traveled by the two wheels

The alignment of the SPCR with the planned path was determined using both angular and positional errors and was updated in real time^43^. The angular error represents the angle difference between the target and current directions, calculated as the absolute difference between them. The positional error is defined as the Euclidean distance between the current and target positions. Further details on the derivatives can be found in the Supplementary section.

**Fig. 5:**
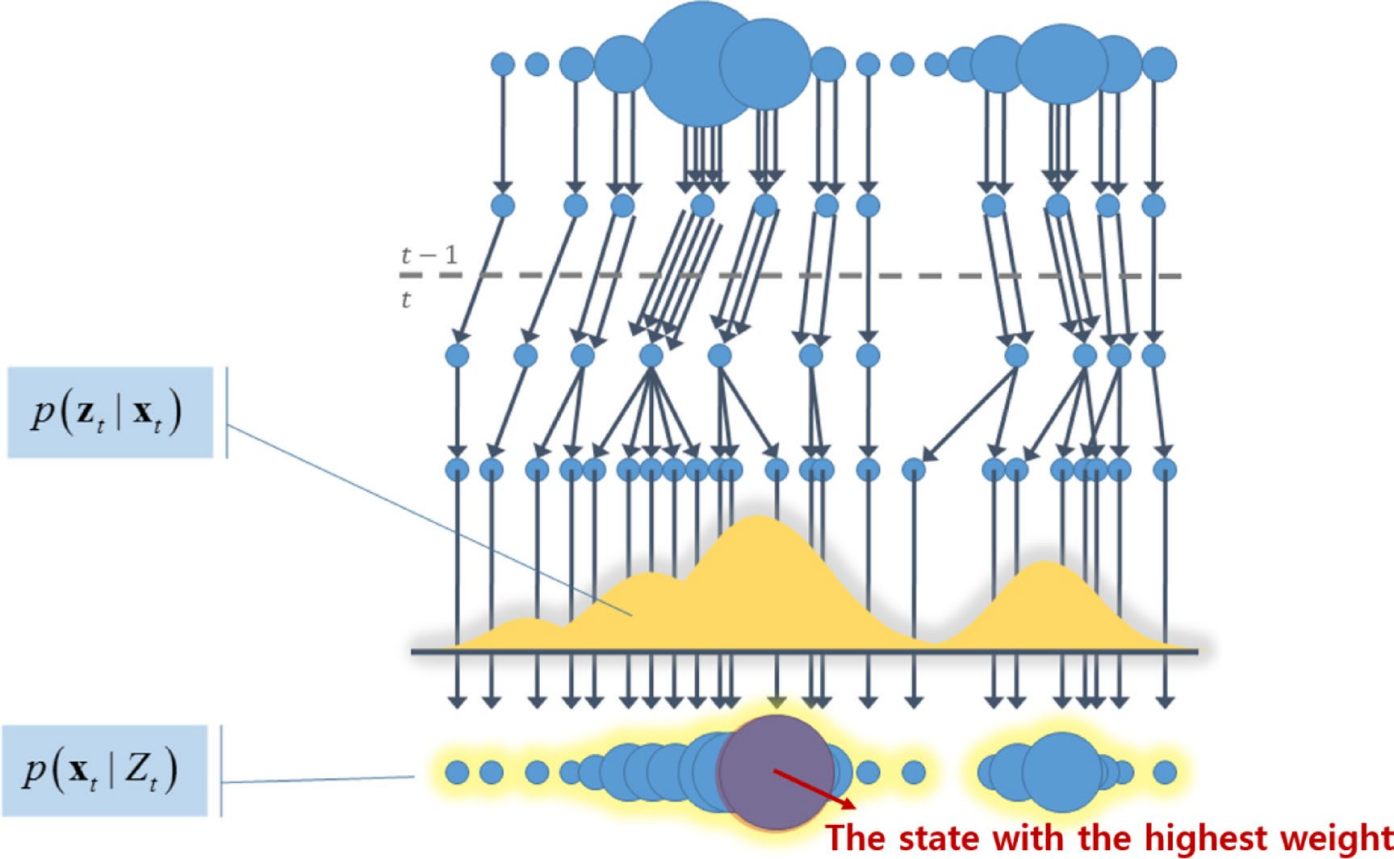
Path for finding likelihood through a particle filter.

The propagation of the particle filter considers the error as a noise term based on the current SPCR configuration. Through the data from the 2D LiDAR, as shown in Fig. 5, information about the surrounding environment is obtained, and the results of this perceptual interaction are referred to as the observation $\:{z}_{t}$. At time t–1, *z*_*t*_ is derived from each predicted state *x*_*t*_, and its likelihood is evaluated. At time t, the state with the highest likelihood weight is selected, and the robot state is updated^44^. The weight at time t is recalculated using the value of $\:{p(x}_{t-1}\:\left|\:{Z}_{t-1)}\right)$ obtained from the pose and weight information. During the transition from t–1 to t, particles with low weights are removed through resampling, and propagation is repeated using wheel odometry noise. This process yields $\:{p(x}_{t}\:\left|\:{Z}_{t)}\right)$ by updating the particles at time t based on the line map and their weights.

### Regional classification

**Fig. 6:**
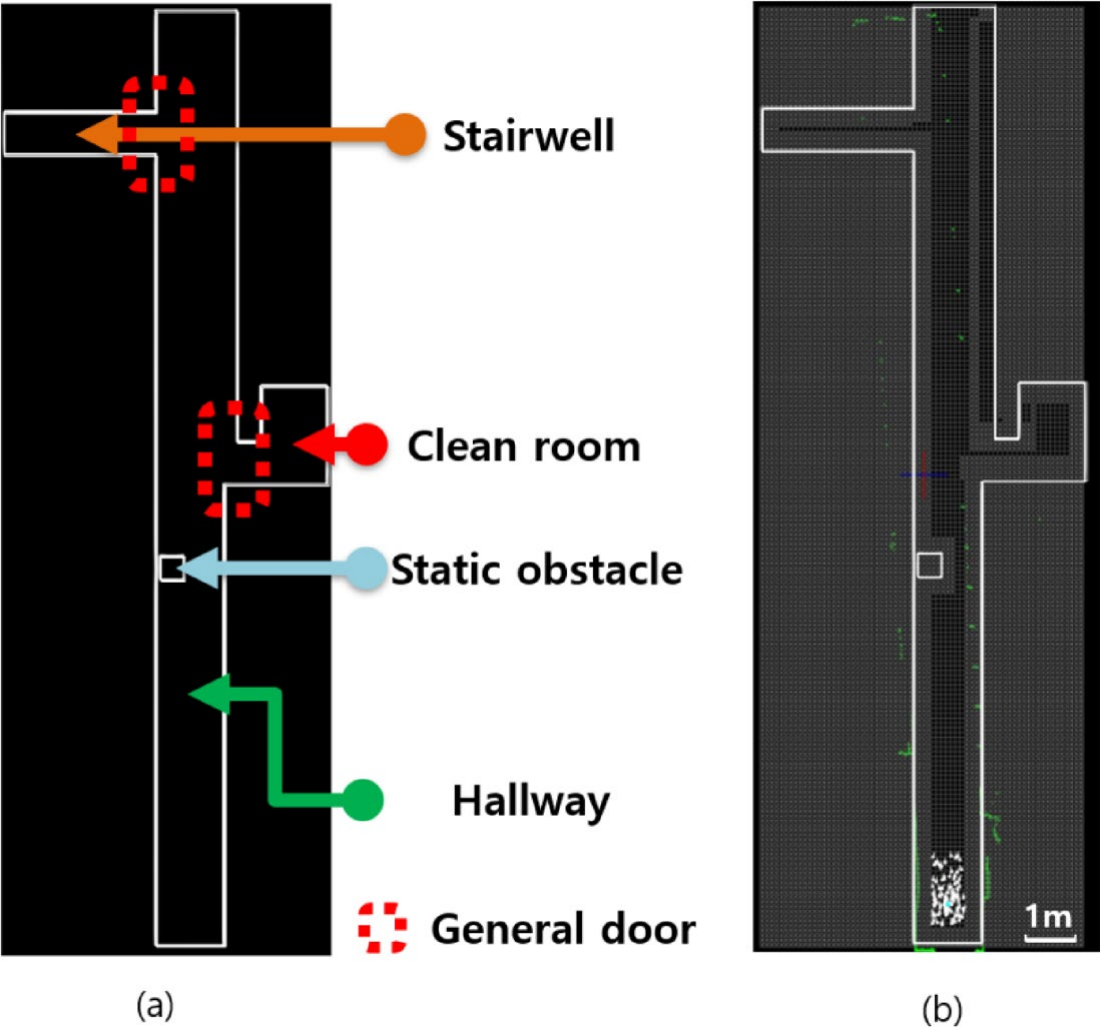
A map guide. (**a**) drawing file in dxf format and (**b**) converting to OGM.

An accurate map of the surrounding environment is essential for navigation tasks such as path planning, surveillance, and coverage. Although real-world environments are dynamic, most mapping approaches assume static conditions. Among these, OGM remains the most widely used approach for representing a given map^45,46^. We configured a space within the hospital as an experimental environment, as shown in Fig. 6. Precise measurements were conducted using a laser displacement sensor to generate a drawing file, which was then converted into the OGM.

**Fig. 7:**
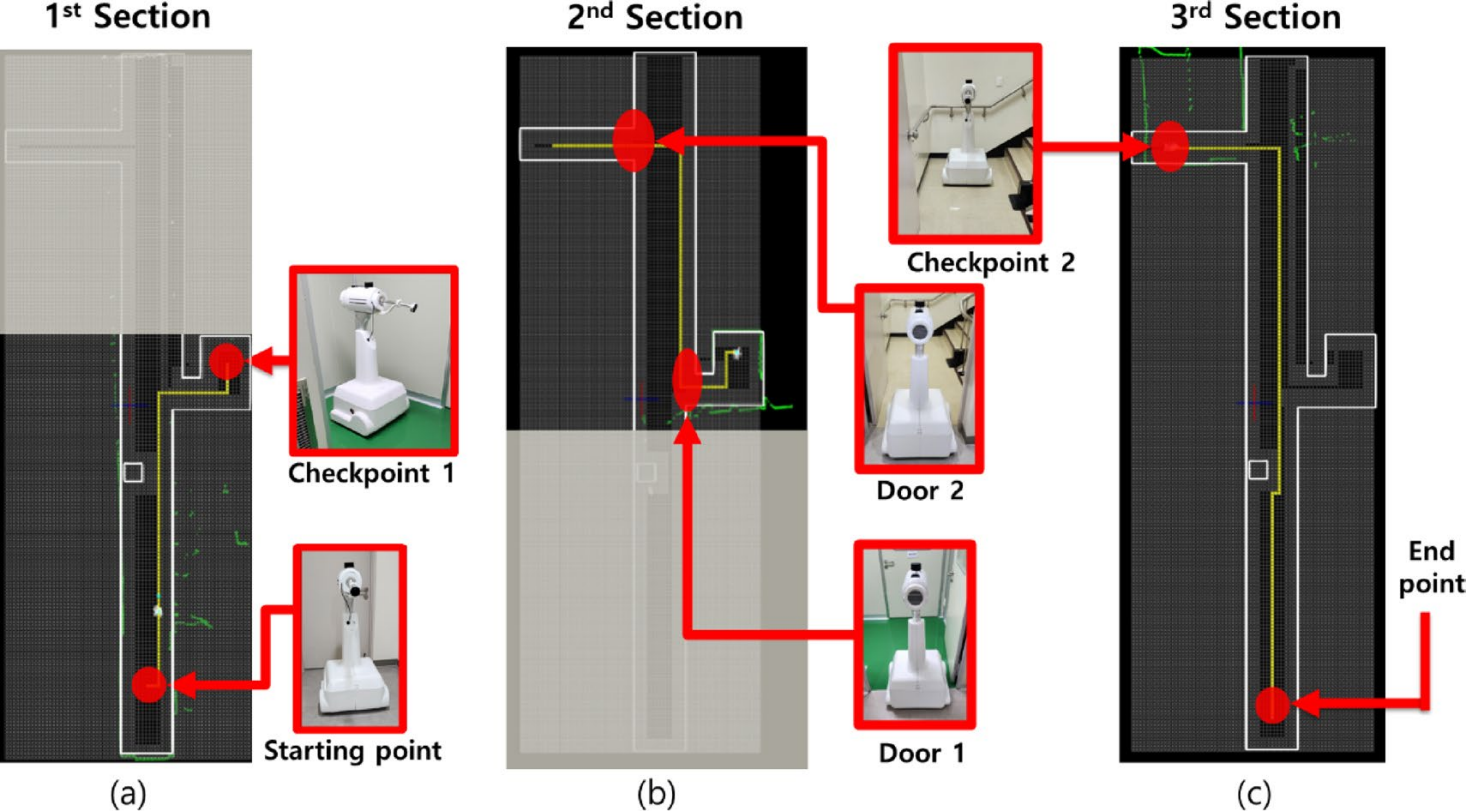
Section classification. (**a**) Section. 1, (**b**) 2, and (**c**) 3.

The mapping was conducted in a hospital hallway, which was divided into three sections for the autonomous driving test, as indicated in Fig. 7. For a standard door was a narrow width of about 90 cm, the driving success rates obtained through ten iterative tests for each section were 60%, 40%, and 60% for Sects. 1, 2, and 3, respectively.

- Section. 1: Initial starting point → Hallway → Obstacle → Door 1 → Checkpoint 1.
- Section. 2: Checkpoint 1 → Hallway → Door 2 → Checkpoint 2.
- Section. 3: Checkpoint 2 → Hallway → Obstacle → starting point.

Where door 1 is a general door, door 2 is stairwell door, checkpoint 1 is the space located approximately in the middle of the entire hallway, and checkpoint 2 is the space connected to the stairwell.

Path lengths averaged 12.94, 14.01, and 18.25 m across sessions, with corresponding traversal times of 93, 122, and 163 s, respectively. The goal of the map modifications was to simplify the path and enable navigation through narrow spaces by ensuring a single open-grid point along the path. Frequent steering was minimized to improve movement accuracy and reduce localization errors, while unnecessary spaces were treated as temporary obstacles to further simplify the path.

## Results

**Fig. 8:**
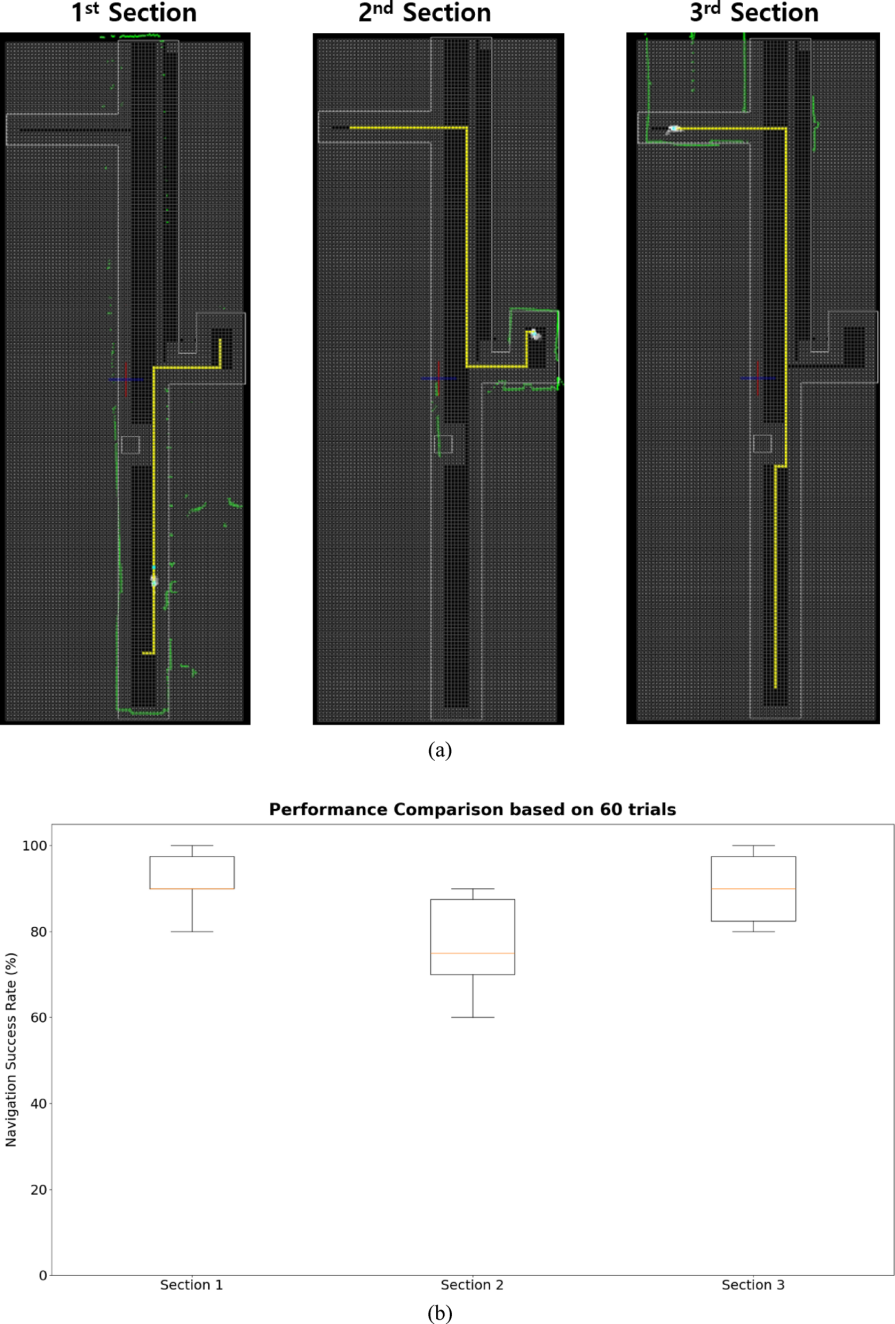
Respective navigation path and success rate. (**a**) navigation path and (**b**) navigation success rate.

Based on this OGM, we validated the navigation success rates for Sects. 1 through 3 shown in Fig. 7. Figure 8a presents the navigation path for each section. The green particles shown in Fig. 8a represented the actual measurement coverage of the 2D LiDAR sensor. The validation was conducted over 60 trials. The navigation success rate obtained from these trials is presented in Fig. 8b. The glass areas demonstrated transmission characteristics, appearing as penetrated regions in certain areas. The most problematic zone for driving success was the narrow doorway. If the robot collided with a wall during its path, it was considered a failure. The OGM modification focused on minimizing sharp turns and increasing the success rate of passing through narrow doorways. Unnecessary movements along the path were also minimized. Figure 8b shows that the navigation success rates after map modification improved by 31.67%, 36.67%, and 30%, respectively.

## Discussion

The environmental model used in this study was designed to closely resemble an actual hospital ward. However, it did not consider dynamic objects, such as moving patients or staff, because patient-care robots are expected to monitor wards during periods of minimal movement, such as at night or when few people are present. Although handling multiple dynamic objects is common for self-driving cars, it is not a typical or desirable scenario for patient-care robots. Considering these slowly moving individuals as static obstacles is therefore not an unrealistic simplification. The LiDAR sensor exhibited no response to lighting variations in dark environments, as it relies on laser-based ranging rather than ambient illumination. Follow-up studies could include dynamic objects in more realistic environments and focus on path optimization in dynamic settings.

WMRs operating in hospital wards must prioritize safety and navigation; therefore, a verified algorithm such as the enhanced D* lite was used instead of the latest untested algorithms. WMRs with large widths face potential collisions in narrow spaces. Guiding the robot to pass through the center of narrow spaces and minimizing its movement before entering these areas helps improve the driving success rate. All collision events were treated as failures during evaluation. In future research, additional safety measures using 3D LiDAR and laser displacement sensors will be implemented to further enhance navigation safety.

Autonomous driving is a basic requirement for the SPCR. A camera attached to the omnidirectional bending part can be used for patient care and monitoring. The robot was designed to detect fallen patients and includes a machine learning algorithm that uses a camera to measure heart rate by extracting facial contours. The implementation of these functions is beyond the scope of this study and will be addressed in future work.

## Conclusion

In this study, we developed and validated an SPCR that improved autonomous driving performance in hospital wards through OGM modifications and enhanced D* lite path planning. By relying only on a single 2D LiDAR sensor, the proposed approach demonstrated that autonomous navigation could be achieved with reduced hardware complexity while maintaining safety and performance. Experimental results confirmed that the driving success rate for passing narrow doorways increased by more than 30% after OGM optimization, and the introduction of virtual obstacle spaces simplified path planning and reduced unnecessary movement. The navigation success rate in Sect. 2 was relatively lower than that of other sections, primarily due to the presence of two doors. These findings indicate that integrating optimized mapping with reliable navigation algorithms effectively reduces collision risks and supports practical deployment of robots in medical environments.

Future work will extend the current framework to account for dynamic objects, such as patients and staff, to increase adaptability to real hospital conditions. Additional sensors, including 3D LiDAR and laser displacement modules, will be integrated to achieve higher robustness and approach nearly 100% navigation success rates. Furthermore, the SPCR platform will be expanded to support advanced patient monitoring functions, such as continuous vital-sign assessment and emergency detection, ultimately enabling a comprehensive robotic assistant capable of safe navigation and proactive patient care.

## Supplementary Information

Below is the link to the electronic supplementary material.

Supplementary Material 1

## Data Availability

The datasets generated in the current study are available from the corresponding author upon reasonable request.
