# Supplementary material for: Development of smart patient care robot with enhanced autonomous navigation through path optimization in hospital wards
Source: Sci Rep. 2026 Jan 22;16:6026. doi: 10.1038/s41598-026-36664-2 (PMC12902103; doi:10.1038/s41598-026-36664-2)
Supplement: Supplementary file 1 — Supplementary Material 1 [file 41598_2026_36664_MOESM1_ESM.docx]

In this study, we calculated it using the enhanced D* lite method as shown in Equation (1).

$rhs\left[ s \right] =$ $\left\{ \begin{aligned} 0, s=s_{goal} \\ \min_{s^{'}\in Succ\left( s \right)} \left( c\left( s,s^{'} \right)+g\left( s^{'} \right) \right), s\neq s_{goal} \end{aligned} \right.(1)$

Here, *Succ(s)* refers to the successor nodes of state *s*, while *c(s,s′)* is the cost of moving from state *s* to *s′*, and *g[s]* is the estimated cost of moving from state s to the goal. *c(s,s′)* is given by the Euclidean distance between the two nodes. *rhs0[s]* represents the minimum one-step lookahead cost of reaching the goal from state *s* and is often better informed than g-values. Based on this formulation, the neighboring state cell with the optimal cost was determined using Equation (2). Incorporating this into the OGM reduced the number of pixels along the navigation path by half. Here, $s_{n+1}$ represents the neighboring state with the minimum cost.

$$s_{n+1}=\arg\min_{s^{'}\in Succ(s_{n})} (c\left( s_{n},s^{'} \right)+g\left( s^{'} \right)) (2)$$
